# Miniature Mobile Robot Using Only One Tilted Vibration Motor

**DOI:** 10.3390/mi13081184

**Published:** 2022-07-27

**Authors:** Renjie Zhu, Yifan Zhang, Hongqiang Wang

**Affiliations:** 1 Shenzhen Key Laboratory of Biomimetic Robotics and Intelligent Systems, Department of Mechanical and Energy Engineering, Southern University of Science and Technology, Shenzhen 518055, China; zhurj@mail.sustech.edu.cn (R.Z.); 11849044@mail.sustech.edu.cn (Y.Z.); 2 Guangdong Provincial Key Laboratory of Human-Augmentation and Rehabilitation Robotics in Universities, Southern University of Science and Technology, Shenzhen 518055, China; 3 Southern Marine Science and Engineering Guangdong Laboratory, Guangzhou 511458, China

**Keywords:** miniature robots, underactuated robots, swarms, mechanism design

## Abstract

In miniature mobile robots, reducing the number of actuators can effectively reduce the size and weight of the robot. However, it is challenging to design a robot with as few actuators as possible without losing good motion performance. This work presented a simple-structured low-cost miniature mobile robot. It is driven by only a single tilted motor and yet is fully capable of being controlled to move forward and turn left or right on the ground. Based on the stick–slip mechanism, the robot’s motion is achieved by interplaying between the centrifugal force generated by the vibration motor tilted on the robot and the friction force of the robot. The robot’s speed can be controlled by regulating the magnitude and the period of the applied voltage. Finally, the robot can translate and rotate on the ground and follow various arbitrary paths. The prototype weighs only 11.15 g, costs $6.35, and is 20 mm in diameter and 25 mm in height. The proposed system is experimentally verified and demonstrates the controllability of the robot by the movement along a straight line, a circle, and more arbitrary paths.

## 1. Introduction

Robots are increasingly being integrated into applications, such as industrial automation, surgical operations, and hazardous environments [1,2,3,4,5,6,7,8,9,10]. Various types of mobile robots with different dimensions are designed [11,12,13,14,15,16,17,18,19,20]. Among them, miniature mobile robots with dimensions of a few centimeters can enter narrow spaces inaccessible to humans for specific tasks [21,22,23]. Benefited by their small size and light weight, they can be easily transported and deployed to restricted locations. Additionally, constrained by size and weight, most of them are deliberately designed to be underactuated to reduce the number of components and energy consumption [24,25,26], and various actuation methods and transmission strategies have been proposed and applied. RoACH robots driven by shape memory alloy coils can move with hexagonal legs and have the capacity to overcome obstacles [20,27]. The HAMR robot consists of multiple piezoelectric actuators driving six legs made of flexure-based spherical five-bar mechanisms [15]. DASH is driven by two motors, one for forwarding motion and the other for steering [19]. The vast majority of robots require two or more actuators for two-dimensional planar motion, for instance, controlling independently of mechanisms on each side of the robot [14,28,29,30], applying the stick–slip locomotion principle [31,32], and having independent DOF of the robot’s legs for steering [15,20]. However, 1STAR is one of the very few robots that uses only one motor to drive the complex gear mechanism to motion [33,34]. This robot is driven by multiple gears to control robot motion by continuously accelerating and decelerating the legs to produce rotation using the compliance disparity between alternate stance legs. In addition, LPMR is driven to move by a piezoelectric actuator through a frequency-controlled bending vibration mode [35]. The downside is that it requires external high-voltage power for driving, limiting its motion capability.

**Figure 1:**
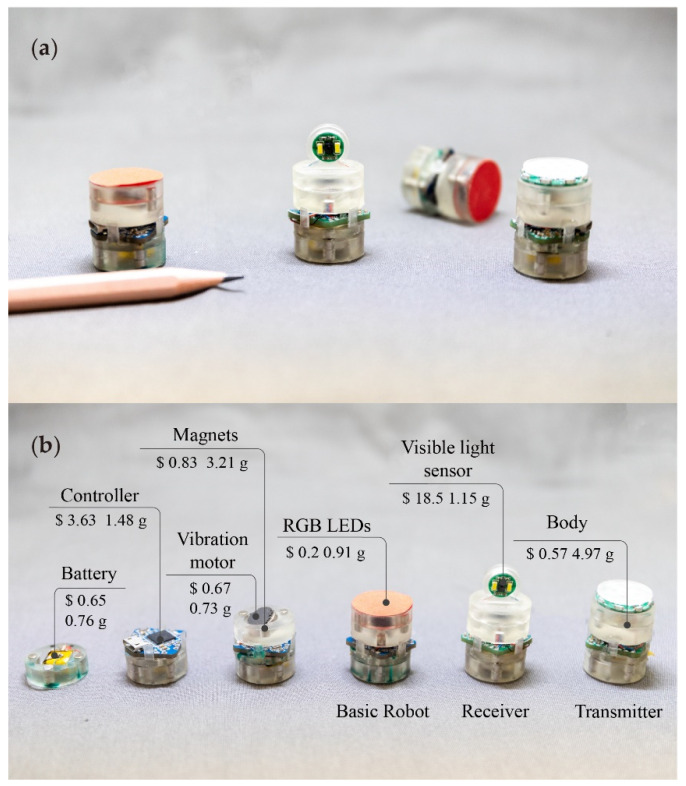
Miniature mobile robots. (**a**) The prototype compared with a pencil as a reference. (**b**) The components of the robot, including some RGB LEDs, a visible light sensor, the robot’s body, a vibration motor, a battery, a controller, and magnets.

In this work, we are committed to designing a miniature mobile robot with as few actuators as possible to reach the ultimate goal (only one). We also aim to design the simplest drive possible, maintaining small size, light weight, and low cost without losing good motion performance. Here, we proposed a miniature mobile robot driven by only a single tilted vibration motor, as shown in Figure 1a. Based on the stick–slip mechanism [36], the robot can translate and rotate simultaneously without any transmission mechanism. The previous famous robot, Kilobot, also employs a similar driving principle [31]. However, a Kilobot has two vertically-placed vibration motors as the power source, and is controlled to move by the vibration amplitude of two independent motors in a differential drive mode. In addition, in the previous work, we designed a single-motor-driven robot called SimoBot [37]. We made the motor tilt by making one of its four legs shorter so that the robot always tilted and swung while moving. The disadvantage is that SimoBot’s body is subject to frictional forces that oscillate during motion, leading to unstable motion and prone to robot tipping. Instead, here, for the stability of the robot motion, we keep the robot upright and place the motor tilted. The centrifugal force from the tilted vibration motor generates torque with friction force and the robot rotates. We can control the rotor speed and the direction of rotation by changing the magnitude of the voltage on the motor. Finally, the robot can be controlled to move along various paths. The detailed locomotion principle is described in the next part.

The robot prototype is composed of four parts: a coin vibration motor, a battery, a microcontroller board, and sensors inside, as shown in Figure 1b. It is only 20 mm in diameter and 25 mm high with an 11.15 g weight, compared with Kilobot’s body size (33 mm in diameter). Furthermore, the cost is low, only $6.35, cheaper than the cost of Kilobot ($14.05), due to these simple commercially available components. With the capability of sensing and communication, this low-cost simple-structured robot offers a possibility for large-scale swarm robot design and manufacturing.

In this paper, at first, we designed a single-motor miniature mobile robot that integrated simple electrical and mechanical components. Next, we built the mathematical model and control strategy for locomotion. After that, we characterized its motor speed and centrifugal force and established the relationship between the voltage and the diameter of circular trajectories. Finally, we verified its controllability by following various paths.

## 2. Robot Design

### 2.1. Mechanical Design

Here, we designed a miniature mobile robot driven by only one vibration motor (Figure 1a). The robot is composed of four layers of cylindrical components (diameter: 20 mm). It is made from Clear Resins (RSF2GPCL04) printed by Form 3+ (Formlabs). As shown in Figure 1b, the battery is stored in the bottom layer (the fourth layer), which is connected to the upper layer by four magnets, two of which supply power to the control board. In addition, to improve the stability of the robot’s operation, the bottom of the fourth layer is designed with four hemispherical protrusions as the robot’s legs in contact with the ground. Next, the third layer is used to hold the control board, which is fixed by four columns connected to the second layer. The vibration motor (Telesky 1030) is fixed at a 30° angle on the second layer. Finally, at the top of the robot (the first layer), depending on the different tasks, various components such as sensors can be attached.

### 2.2. Locomotion

As mentioned above, the robot we proposed here is based on the stick–slip mechanism using only one tilted motor. The advantage of this mechanism is that no transmission device is required. The eccentric mass in the vibration motor, driven by the alternating electromagnetic field, generates centrifugal force. If the vibration motor is placed horizontally on the robot, the centrifugal force on the robot is equal in magnitude in different directions. Then, the robot will move forward and backward at the same distance and, ultimately, it does not produce any displacement (i.e., the average velocity is zero). However, if the motor is tilted, it causes a difference in the vertical component of the centrifugal force between forward and backward, causing the robot to translate. At the same time, the centrifugal force and the frictional force generate a torque that rotates the robot. Thus, if the motor is kept rotating at a constant speed, the robot runs on a circular path. Detailed kinematic and dynamics analysis is performed in the next section.

### 2.3. Communication and Sensing

To give our robots the ability to sense the orientation of their nearby robots as well, we transmitted information via visible light. Specifically, we designed a couple of robots: Transmitter and Receiver. On the top of Transmitter, we installed eight blue LEDs, evenly distributed on a 360-degree circle. On the other hand, a visible light sensor (AS7341) that can identify eight channels of visible light (identifying the blue light, 435–455 nm, in this experiment) is placed on the top of Receiver. Depending on the power of the LEDs and the ambient light, the maximum communication distance between them can reach 30 cm (about 15 times the diameter of the robot).

### 2.4. Electrical Circuit

**Figure 2:**
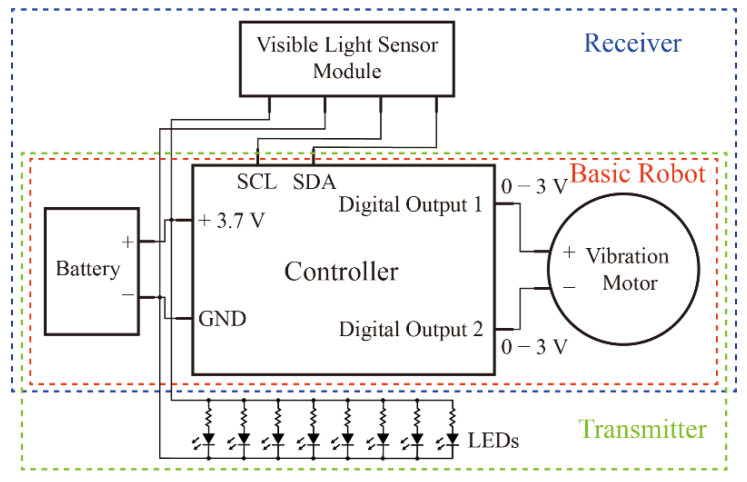
Schematic diagram of the control circuit.

Here, the robot has a 3.7 V 50 mAh lithium-ion chargeable battery (Bolang 501012) to power the entire system, as shown in Figure 2. The controller used is an Atmega328 microprocessor and its two digital outputs with pulse width modulation (PWM) pins used for controlling the speed of the vibration motor. Turning the digital outputs on and off controls the motor’s operation and stopping, respectively. When the voltage generated by digital output 1 is higher than digital output 2, the rotor rotates clockwise, and vice versa, the rotor rotates counterclockwise. In addition, for Receiver, the visible light sensor uses I^2^C serial communication protocol for communication with the controller. Furthermore, the controller has an additional four output pins available for accessory sensors and actuators, which may be useful for complex tasks.

### 2.5. Cost

As shown in Figure 1b, the cost of each robot can be divided into six categories: main structure (i.e., four-layer-cylindrical assembly) is $0.57, communication and sensing module (including LEDs, visible light sensor, and their supporting circuit components) is $18.70, battery is $0.65, controller is $3.63, and vibration motor is $0.67. The overall cost is only $6.35 for the basic robot and $24.85 for Receiver, much cheaper than most other miniature robots [11,28,31].

## 3. Modeling

### 3.1. Kinematics and Dynamics Analysis

**Figure 3:**
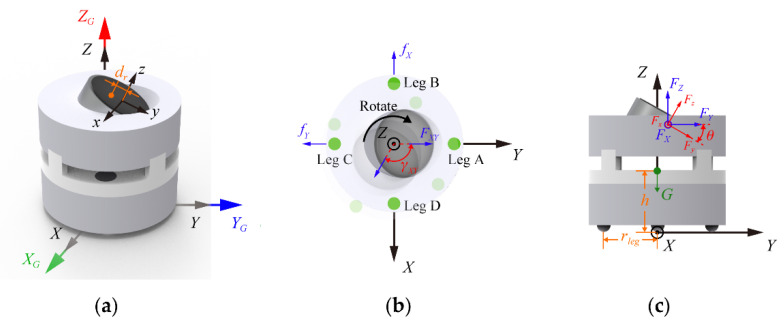
Definition of parameters and load distribution. In the coordinate system XYZ, the line from leg B to D is the X axis, the center of the legs is the origin, and the XY plane is horizontal. (**a**) Cartesian coordinate systems in robot modeling. The forces on the robot resulting in rotating around the Z-axis in (**b**) and translation on the XY plane in (**c**).

As shown in Figure 3a, there are three coordinate systems, including a global one, $X_{G}Y_{G}Z_{G}$ and two local ones, xyz (on the vibration motor) and XYZ (on the robot body).

The centrifugal force from the rotor is $F=m_{r}d_{r}\omega_{r}{}^{2}$, where $d_{r}$ is the distance between the gravity center of the vibration motor and the z-axis, $m_{r}$ is the mass of eccentric rotor, and $\omega_{r}$ is the angular speed of the vibration motor. Other parameters in this paper are listed in Table A1. The angular displacement of the vibration motor at time *t*, as shown in Figure 3b, becomes $\gamma_{XY}=\omega_{r}t$.

Therefore, the centrifugal force in the coordinate system XYZ, as shown in Figure 3c, can be obtained by $F_{X}=-F\sin\gamma_{XY}$, $F_{Y}=F\cos\gamma_{XY}\cos\theta$, and $F_{Z}=F\cos\gamma_{XY}\sin\theta$, where θ is the angle of the tilted motor. $F_{XY}=\left[F_{X},F_{Y}\right]$ is the component force obtained by decomposing the centrifugal force F on the XY plane. In time *t*, $F_{XY}$ goes from [0,cosθ] initially to $\left[-\sin\gamma_{XY},\cos\gamma_{XY}\cos\theta \right]$, as shown in Figure 3b. The angle between these two vectors $\gamma_{XY}$ is the rotation angle of the robot. Thus, we can obtain it by $\left|\cos\gamma_{XY}\right|=\frac{\cos\theta }{\sqrt{\tan^{2}\gamma_{XY}+\cos^{2}\theta }}$ and $\left|\sin\gamma_{XY}\right|=\frac{\tan\theta }{\sqrt{\tan^{2}\gamma_{XY}+\cos^{2}\theta }}$.

When the robot moves on the XY plane, the friction force is $f_{XY}=\mu \left(mg-F_{Z}\right)$, where μ is the coefficient of friction between the robot and ground and m is the mass of the robot. As shown in Figure 3b, the robot moves on the XY plane, subjected to both the centrifugal force and the friction force. The direction of the friction force varies as the variation of the movement state of the robot. When the robot moves (i.e., $v_{X}$ and $v_{Y}\neq 0$), the direction of the friction force is opposite to the direction of speed. The accelerations of the robot in the X and Y directions can be obtained by, respectively:

(1) $$a_{X}=\frac{F_{X}-sig\left(v_{X}\right)\left|-f_{XY}\sin\gamma_{XY}\right|}{m},$$

(2) $$a_{Y}=\frac{F_{Y}-sig\left(v_{Y}\right)\left|f_{XY}\cos\gamma_{XY}\right|}{m}.$$

While the robot is at rest, if the centrifugal force is greater than the friction force, the friction force is the reaction force of the centrifugal force (same magnitude but opposite direction). The accelerations of the robot in the X and Y directions are:

(3) $$a_{X}=\frac{F_{X}-sig\left(F_{X}\right)\left|-f_{XY}\sin\gamma_{XY}\right|}{m},$$

(4) $$a_{Y}=\frac{F_{Y}-sig\left(F_{Y}\right)\left|f_{XY}\cos\gamma_{XY}\right|}{m}.$$

In addition, the robot can also rotate around the Z-axis as it is subjected to the moments both from the rotor and the friction in the XY plane. Similar to the acceleration in X and Y directions, when the robot rotates (i.e., $\omega_{Z}\neq 0$), the direction of the friction force is opposite to the direction of rotation, the angular acceleration of the robot motion $\alpha_{Z}$ can be obtained as:

(5) $$\alpha_{Z}=\frac{F_{XY}d\sin\theta -sig\left(\omega_{Z}\right)\left|f_{XY}\right|r_{leg}}{J_{Z}},$$

where $J_{Z}$ is the moment of inertia of the robot around the Z-axis, d is the distance between the origin of the coordinate system xyz and XYZ, and $r_{leg}$ is the distance between the robot’s legs and the Z-axis. When the robot is stationary and the centrifugal force is greater than the friction force, the direction of the friction force is opposite to the centrifugal force. In other cases, the angular acceleration is zero. Therefore, the angular acceleration of the robot motion $\alpha_{Z}$, is:

(6) $$\alpha_{Z}=\frac{F_{XY}d\sin\theta -sig\left(F_{XY}\right)\left|f_{XY}\right|r_{leg}}{J_{Z}}.$$

### 3.2. Trajectory Modeling

According to $a_{X}$, $a_{Y}$, $\alpha_{Z}$ obtained from the above-mentioned equations. After ith iteration of incremental time (∆t), the robot’s velocity, displacement, and angular angle can be obtained as $∆X\left(i\right)=v_{X}\left(i\right)∆t=\left[v_{X}\left(i-1\right)+a_{X}\left(i-1\right)∆t\right]∆t$, $∆Y\left(i\right)=v_{Y}\left(i\right)∆t=\left[v_{Y}\left(i-1\right)+a_{Y}\left(i-1\right)∆t\right]∆t$, and $∆\varphi_{Z}\left(i\right)=\omega_{Z}\left(i\right)∆t=\left[\omega_{Z}\left(i-1\right)+\alpha_{Z}\left(i-1\right)∆t\right]∆t.$ Thus, the position and angle of the robot in the coordinate system $X_{G}Y_{G}Z_{G}$ are:

(7) $$X_{G}\left(i\right)=X_{G}\left(i-1\right)+∆X\left(i\right)\cos\left(∆\varphi_{Z}\left(i\right)\right)+∆Y\left(i\right)\sin\left(∆\varphi_{Z}\left(i\right)\right),$$

(8) $$Y_{G}\left(i\right)=Y_{G}\left(i-1\right)+∆Y\left(i\right)\cos\left(∆\varphi_{Z}\left(i\right)\right)-∆X\left(i\right)\sin\left(∆\varphi_{Z}\left(i\right)\right),$$

(9) $$\varphi_{Z}=\sum^{\;}∆\varphi_{Z}.$$

**Figure 4:**
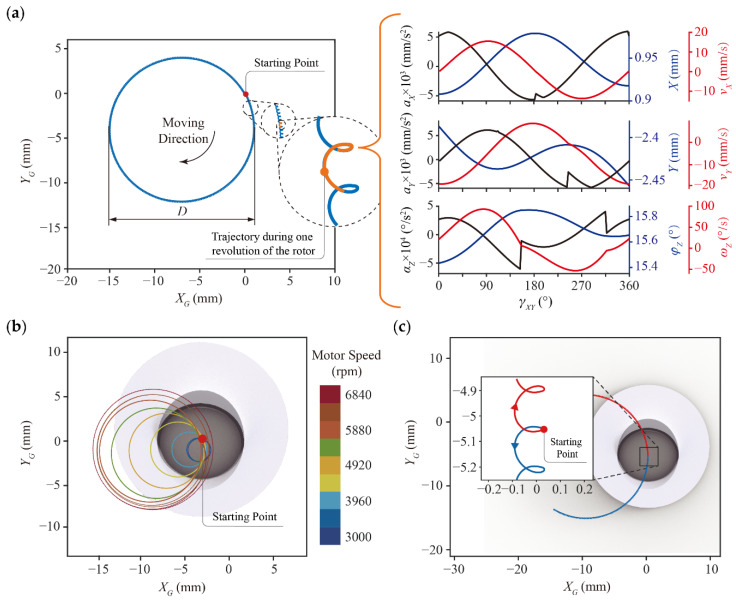
The movement of the robot on the $X_{G}Y_{G}$ plane. (**a**) The trajectory circle generated by the robot while the rotor continuously spins at a constant speed. The orange spiral line is the trajectory during one revolution of the rotor. There is some parameter variance including the acceleration, velocity, and displacement on both X-axis and Y-axis, and the angular acceleration, angular velocity, and rotational angle along the Z-axis. (**b**) The corresponding trajectories of the robot under different motor speed. (**c**) The blue and red arcs represent that the robot’s motor is driven by positive and negative voltage and the robot rotates in counterclockwise and clockwise directions, respectively.

With the aforementioned equations, we can estimate the trajectory of the robot (Figure 4). The parameters used in the equations are consistent with those of the robot prototype (Table A2). Assuming that the vibration motor is subjected to a positive voltage, the trajectory of the robot forms a short spiral arc, shown as the orange curve in Figure 4a, after the rotor rotates counterclockwise for one revolution ($\gamma_{XY}$ from 0° to 360°). If the voltage is kept constant and the rotor continues to rotate repeatedly, the spiral arcs are connected in series with each other, resulting in a clockwise circular trajectory with diameter D. Each revolution of the rotor produces a trajectory that is identical in shape, due to the robot moving on the XY plane is subjected to both centrifugal and friction forces that vary continuously from period to period. In addition, we analyzed either one of the revolution periods and the results show that when the robot’s velocity is equal to zero, its acceleration and velocity profiles generate discontinuities based on Equations (1)–(6). In the X-direction, for example, when $\gamma_{XY}$ is approaching 180°, the robot brakes to rest and then accelerates again. In this process, the net force of centrifugal force and friction force on the robot changes from $F_{X}+\left|f_{X}\right|$ to $F_{X}-\left|f_{X}\right|$, thus causing its acceleration to be discontinuous. This similar phenomenon occurs for translation in the Y-direction and rotation along the Z-axis. When the rotor speed rises, the trajectory circle becomes larger (Figure 4b). Furthermore, when the voltage is negative, the rotor reserves its rotational direction and the robot eventually moves in a counterclockwise circular trajectory (red curve in Figure 4c).

**Figure 5:**
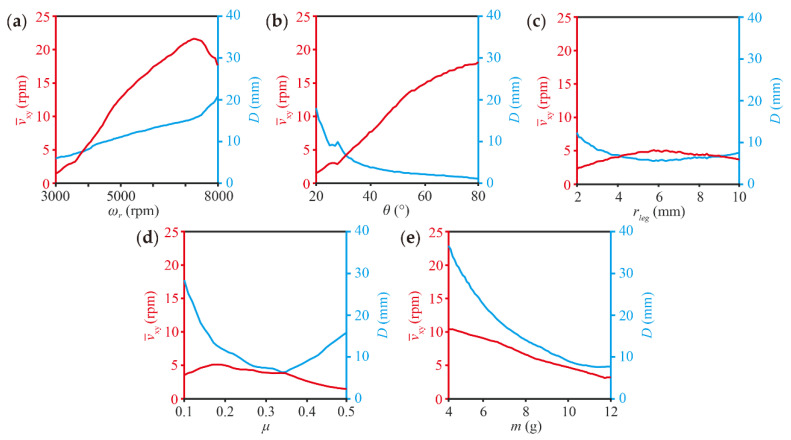
Effects of different parameters on robot motion performance, including (**a**) the motor speed, (**b**) the initial inclination angle, (**c**) the horizontal distance between the legs, (**d**) the coefficient of friction, and (**e**) robot mass.

Here, we analyzed the parameters, including the motor speed ($\omega_{r}$), initial inclination angle (θ), the horizontal distance of the legs ($r_{leg}$), the coefficient of friction (μ), and the robot mass (m) based on the equations mentioned above to optimize the design and control of the robot. As shown in Figure 5, higher $\omega_{r}$ increases D. When $\omega_{r}$ is about 6000 rpm, the robot’s speed reaches the peak. The greater θ decreases D but increases average linear speed and the same trend is also seen in $r_{leg}$. In addition, the linear speed approaches peak when μ is approximately 0.3, but larger μ results in smaller D. As the robot mass increases, both speed and trajectory diameter remain decreasing. In summary, the size selection of θ and $r_{leg}$ requires finding a balance between diameter and translation speed. On another aspect, the lightest possible robot’s mass has a better effect on the robot’s motion performance.

## 4. Motion Strategy

According to the previous section, the robot’s trajectory is only a circle when its motor is under a constant voltage, so it cannot be directly controlled to move along complex paths. To give it the ability to follow a complex path accurately, we converted the path into some simple paths suitable for the robot. In other words, we cut the complex path into some arcs of different lengths and diameters. After that, the robot can be controlled sequentially to follow the specified arcs and stitch them together into a complete trajectory.

**Figure 6:**
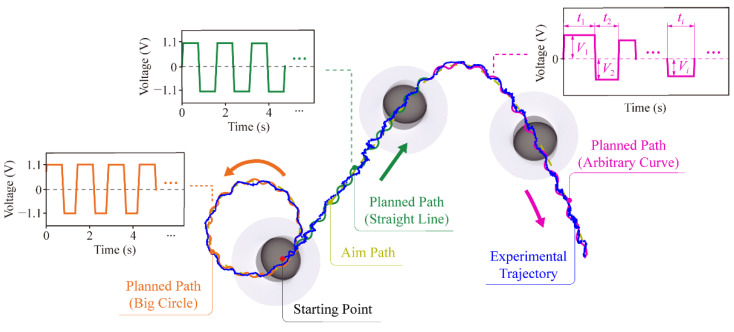
The path of the robot while fitting a large circle, a straight line, and an arbitrary curve, respectively.

Here, as shown in Figure 6, we designed a complex path containing a big circle, a straight line, and an arbitrary curve to illustrate our motion strategy.

(1) At first, we split the aim path (light green dashed line) into arcs of different diameters and lengths that the robot can follow.

(2) The robot moves counterclockwise during the big circle path (orange line). The aim path is divided into some tangent arcs and the inner arcs are shorter than the outer arcs. The straight line (dark green line) can be planned as arcs of equal length to connect. Furthermore, the arbitrary curve (purple line) is made of arcs (at different diameters and lengths).

(3) After we connected the arcs to fit the aim path, we can calculate the length of each arc according to the Equations (7) and (8). Then we can estimate the practical angle and determine the voltage and duration of the motor for each arc. The motor is sequentially controlled by specified voltage and time for arbitrary curves. According to the kinematic and kinetics model, the diameter of the arc is determined by the driving voltage on the motor. In addition, the length of the arc is controlled by the duration of the voltage applied to the motor. For example, to follow the big circle path, as shown in the orange line in Figure 6, the motor is powered by +1.1 V (0.8 s) for following the outer arcs and −1.1 V (0.6 s) for the inner arcs, repeatedly. When the robot follows the straight line, the motor is powered by +1.1 V (0.8 s) and then −1.1 V (0.8 s), repeatedly.

However, since the robot’s experimental trajectory is a combination of arcs of different lengths, the difference between the aim path and the planned path always exists. The error can be reduced by increasing the arcs of the planned path. Due to the radius of the arc, there is a limitation when the aim path has a sharp turn. In addition, increasing the number of arcs and reducing their lengths may cause the robot to switch the direction of motion frequently. The experimental trajectory of the robot has a larger error due to the unevenness of the test platform.

## 5. Experiments 

### 5.1. Performance of the Motor

**Figure 7:**
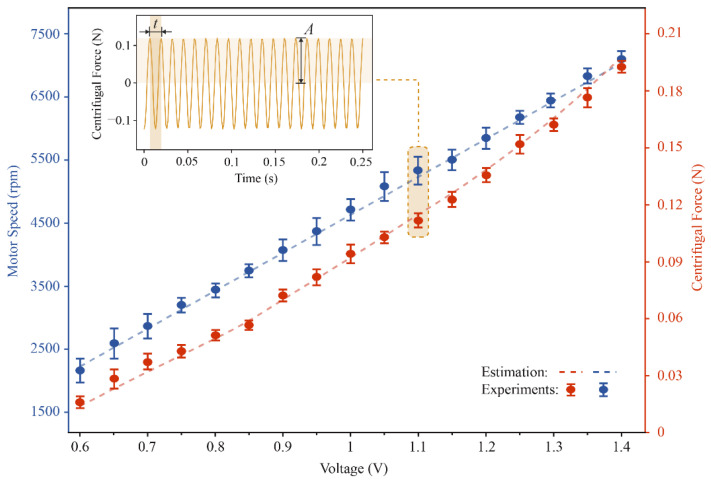
The motor speeds and centrifugal force on different voltages. The centrifugal force of the motor while the motor is powered on 1.1 V.

As aforementioned, the rotor speed is an important parameter that affects the robot’s motion. To measure the speed and centrifugal force of the vibration motor, we connected the vibration motor to a load cell (FUTEK LSB200). In our experiments, we set the voltage between 0.6 V–1.4 V, as shown in Figure 7, and the data obtained by the sensor vary with time as a periodic sinusoidal function, where the frequency represents the rotor’s speed. The amplitude represents the magnitude of the centrifugal force generated by the rotor. In addition, as the voltage increases, the speed increases linearly, while the centrifugal force of the vibration motor is quadratically related to the speed. By fitting the centrifugal force data, the equation for the relationship between centrifugal force and rotational speed can be obtained as $F=3.91\times 10^{-7}\omega^{2},$ where the product of the mass of the eccentric rotor and its eccentric distance is $3.91\times 10^{-7}\;\mathrm{kg}\cdot m$.

### 5.2. Following Circular Trajectories

**Figure 8:**
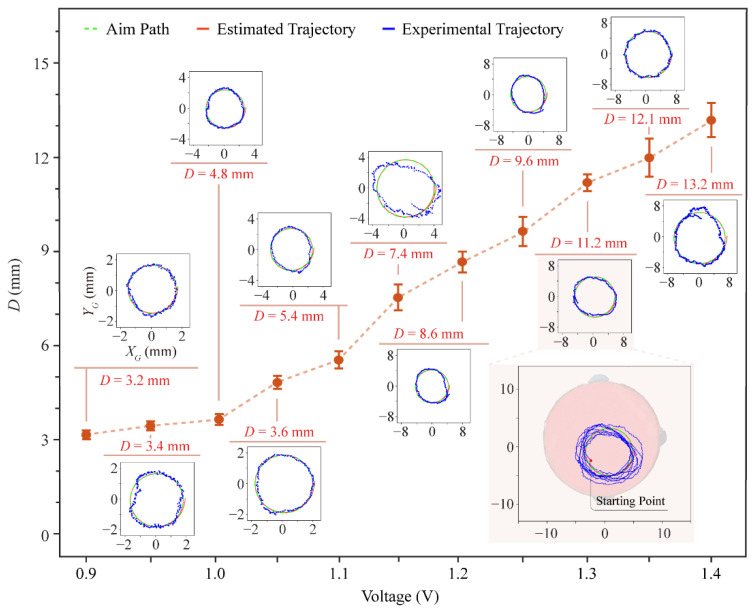
The diameter of the robot’s running trajectories on different voltages.

The robot’s circular trajectory was tracked by a camera above by identifying the center of the red marker (red circular paper) attached to the robot’s top. The robot was driven by a +1.3 V DC voltage and its corresponding trajectory of running nine loops in the experiment, as shown in Figure 8. As the voltage rises, the diameter of the circular trajectory increases (see Figure 8 and Video S1). The results of the experimental and theoretical calculations are approximately the same in terms of trend. Its motion speed increased from 3 mm/s (0.9 V), 6.18 mm/s (1.1 V), 8.19 mm/s (1.2 V) to 9.72 mm/s (1.4 V). These deviations are mainly caused by the rough surface of the testbed and variable friction, and errors can be improved in the future by closed-loop control.

### 5.3. Following Arbitrary Trajectories

Based on the motion strategy mentioned, the robot can be controlled to follow various paths by connecting the small arcs. Suppose the voltage of vibration motor is repeatedly flipped at the same period and the voltage amplitude is kept constant; in which case, the robot can move short arcs of the same length along clockwise and counterclockwise alternately and, finally, generate a straight line. When the duration of the voltage applied to the motor is different, the robot’s trajectory is no longer a straight line but a curve. The radius of the curve decreases with the larger difference between the period of positive voltage and negative voltage on the motor.

**Figure 9:**
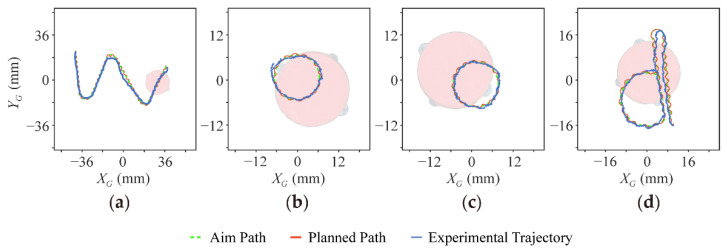
(**a**–**d**) The trajectories of the robot fitting all the letters of WOOD (in honor of Professor Robert J. Wood).

The robot can follow a more arbitrary path based on a similar strategy. As shown in Figure 9 and Video S2, we used our robot to draw the letters of “WOOD” (in honor of Professor Robert J. Wood, in the Special Issue “Microrobotics: A Commemorative Issue in Honor of Professor Robert J. Wood”). For instance, to generate a trajectory pattern for the letter “W” in the linear segment, the motor was powered by +1.0 V (0.5 s) and then −1.0 V (0.5 s) repeatedly for seven loops. When turning through the corner, it was powered by +1.0 V for 2 s. 

In addition, the path of “d” is composed of a semicircle and two straight lines. Similarly, the robot was driven by +1.0 V (0.6 s) and then −1.0 V (0.4 s). After repeating these steps for 10 s, the robot entered a straight line. In particular, the robot performed a 180-degree turnaround when given −0.7 V for 2.4 s. All alphabets are assembled from arcs suitable for the robot to walk, showing its great controllability without any feedback.

### 5.4. Commuication and Sensing

**Figure 10:**
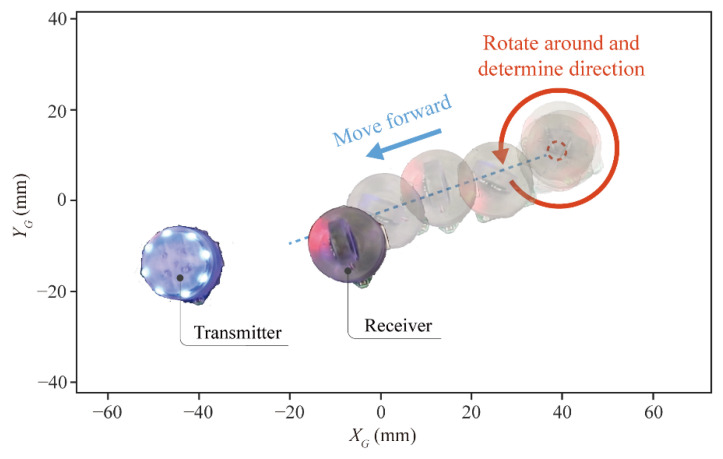
Process of Receiver approaching Transmitter.

In the above section, we verified a single robot’s motion capability and strategy. This section will discuss the ability to communicate and sense between multiple robots. Here, we designed two robots: Transmitter and Receiver. Transmitter’s top-mounted eight blue LEDs emit light signals outward. Receiver has a visible light sensor mounted on the top that senses the light intensity of different wavelengths of visible light. Its task is to detect the light signal, determine Transmitter’s location, and finally approach it. The distance between them is about 114 mm. As shown in Figure 10 and Video S3, the whole task can be divided into three steps as follow:

**Step 1:** Record the maximum light intensity during the entire rotation.

Receiver rotated around and detected all blue light intensity at 360°. Only the higher intensity will be recorded during rotating compared with the previous signal intensity. Finally, the largest intensity will be recorded.

**Step 2:** Determine the moving direction.

During this period, Receiver continued to rotate, detected the light intensity value in the current direction, and compared it with the previously recorded maximum light intensity.

**Step 3:** Approaching Transmitter.

Receiver moved straight towards Transmitter, taking about 20 s to reach the goal.

## 6. Conclusions and Discussion

In this work, we designed a compact (20 × 20 × 25 mm), lightweight (11.15 g), low-cost ($6.35), miniature mobile robot, which only uses one tilt-placed vibration motor for motion. We built the kinematics and kinetics models, analyzed and optimized the parameters, and proposed a control strategy. Experimental results show that when the vibration motor is powered by a constant DC voltage, the robot’s trajectory is a circle. The diameter of the circle is related to the given voltage. We can successfully control the robot following on various aim paths, such as a straight line, a big circle, and any arbitrary curves, by stitching the arcs suitable for the robot to run sequentially.

Although the control in this work is currently open loop, with the addition of sensors and appropriate position control algorithms, the robot can improve the accuracy of path tracking through closed-loop control. In addition, we installed sensors on the robots to enable communication between two robots, providing a possibility for cooperation and communication among multiple robots. Especially in some narrow spaces, the miniature mobile robots will be able to reach places that are generally inaccessible to humans, using their sensors to assist humans in their exploration work. Further, in the case of a cluster of multiple miniature robots, each robot can communicate with each other to achieve a division of labor and cooperation, which has advantages in special and complex environments.

## Data Availability

Not applicable.

## Supplementary Materials

The following are available online at https://www.mdpi.com/article/10.3390/mi13081184/s1, Video S1: Running circle paths, Video S2: Running on different paths, and Video S3: Communication and sensing.

## Appendix A

**Table A1:** List of symbols.

Symbols	Description
x, y, z	Coordinate system 1 (on the vibration motor)
X, Y, Z	Coordinate system 2 (on the robot body)
$X_{G},\;Y_{G},\;Z_{G}$	Coordinate system 3 (the global coordinate system)
$m_{r}$	The mass of the rotor and eccentric mass
$\omega_{r}$	The angular speed of the vibration motor
$d_{r}$	The distance between the rotor’s center of gravity and the z axis
F	The centrifugal force generated by the rotor
θ	The tilt angle of the motor
$F_{x}$ $,\;F_{y}$ $,\;F_{z}$	The components of the centrifugal force F in the x, y, and z directions
$F_{X}$ $,\;F_{Y}$ $,\;F_{Z}$	The components of the centrifugal force F in the X, Y, and Z directions
$F_{XY}$	The components of the centrifugal force F in the XY plane
$f_{XY}$	The friction force in the XY plane
$\gamma_{XY}$	$\mathrm{The}\;\mathrm{angle}\;\mathrm{between}\;F_{XY}$ and the Y axis
μ	The coefficient of friction between the robot legs and the ground
m	The mass of the robot
g	Gravitational acceleration
d	The distance between the origins of coordinate system 1 and 2
h	The distance between the robot gravity center and the origin of coordinate system 2
$f_{X}$ $,\;f_{Y}$	$\mathrm{The}\;\mathrm{components}\;\mathrm{of}\;\mathrm{the}\;\mathrm{friction}\;\mathrm{force}\;f_{XY}$ in the X and Y directions
$a_{X}$ $,\;a_{Y}$	The acceleration of the robot in X and Y directions
$\alpha_{Z}$	The angular acceleration of the robot around the Z axis
$J_{Z}$	The moment of inertia of the robot around the Z axis
$r_{leg}$	The distance between the robot’s leg and the Z axis at the initial status
∆t	The time step for each iteration
$v_{X}$ $,\;v_{Y}$	The linear speed of the robot in the X and Y directions
$\omega_{Z}$	The angular speed of the robot around Z axis
∆X , ∆Y	The displacement of the robot during each iteration in the X and Y directions
$∆\varphi_{Z}$	The angular angle of the robot around the Z axis during each iteration
$X_{G}$ $,\;Y_{G}$	The position of the robot in the global coordinate system

**Table A2:** Parameters used in the estimation.

**Parameters**	**Value**
$m_{r}$	$2.5\times 10^{-4}$ kg
$d_{r}$	$1.54\times 10^{-3}$ m
$\omega_{r}$	3600 rpm
θ	30°
μ	0.3
m.	$11.15\times 10^{-3}$ kg
h	$2.5\times 10^{-3}$ m
$J_{Z}$	$4.09\times 10^{-7}$ $\mathrm{kg}\cdot m^{2}$
$r_{leg}$	0.01 m
∆t	$10^{-4}$ s

## References

[1] Suri S., Jain A., Verma N., Prasertpoj N. SCARA Industrial Automation Robot. Proceedings of the International Conference on Power Energy, Environment and Intelligent Control (PEEIC).

[2] Al Khawli T., Anwar M., Alzaabi A., Sunda-Meya A., Islam S. Machine learning for robot-assisted industrial automation of aerospace applications. Proceedings of the IEEE International Conference on Systems, Man, and Cybernetics (SMC).

[3] Mohammadi Amin F., Rezayati M., van de Venn H.W., Karimpour H. (2020). A Mixed-Perception Approach for Safe Human-Robot Collaboration in Industrial Automation. Sensors.

[4] Freschi C., Ferrari V., Melfi F., Ferrari M., Mosca F., Cuschieri A. (2013). Technical review of the da Vinci surgical telemanipulator. Int. J. Med. Robot..

[5] Yang C., Guo S., Bao X. (2022). An Isomorphic Interactive Device for the Interventional Surgical Robot after In Vivo Study. Micromachines.

[6] Wang W., Li J., Wang S., Su H., Jiang X. (2016). System design and animal experiment study of a novel minimally invasive surgical robot. Int. J. Med. Robot..

[7] Petereit J., Beyerer J., Asfour T., Gentes S., Hein B., Hanebeck U.D., Kirchner F., Dillmann R., Götting H.H., Weiser M. ROBDEKON: Robotic systems for decontamination in hazardous environments. Proceedings of the IEEE International Symposium on Safety, Security, and Rescue Robotics (SSRR).

[8] Lee C.H., Kim S.H., Kang S.C., Kim M.S., Kwak Y.K. (2003). Double-track mobile robot for hazardous environment applications. Adv. Robot..

[9] Luk B.L., Cooke D.S., Galt S., Collie A.A., Chen S. (2005). Intelligent legged climbing service robot for remote maintenance applications in hazardous environments. Rob. Auton. Syst..

[10] Seward D., Pace C., Agate R. (2007). Safe and effective navigation of autonomous robots in hazardous environments. Auton. Robot..

[11] Mondada F., Bonani M., Raemy X., Pugh J., Cianci C., Klaptocz A., Magnenat S., Zufferey J.C., Floreano D., Martinoli A. The e-puck, a robot designed for education in engineering. Proceedings of the 9th Conference on Autonomous Robot Systems and Competitions.

[12] Li S., Batra R., Brown D., Chang H.D., Ranganathan N., Hoberman C., Rus D., Lipson H. (2019). Particle robotics based on statistical mechanics of loosely coupled components. Nature.

[13] Erdem E.Y., Chen Y.M., Mohebbi M., Suh J.W., Kovacs G.T.A., Darling R.B., Bohringer K.F. (2010). Thermally Actuated Omnidirectional Walking Microrobot. J. Microelectromech. Syst..

[14] Kim S., Clark J.E., Cutkosky M.R. (2016). iSprawl: Design and Tuning for High-speed Autonomous Open-loop Running. Int. J. Rob. Res..

[15] Baisch A.T., Ozcan O., Goldberg B., Ithier D., Wood R.J. (2014). High speed locomotion for a quadrupedal microrobot. Int. J. Rob. Res..

[16] Arvin F., Samsudin K., Ramli A.R. (2009). Development of a miniature robot for swarm robotic application. Int. J. Electr. Comput. Eng..

[17] Goldberg B., Zufferey R., Doshi N., Helbling E.F., Whittredge G., Kovac M., Wood R.J. (2018). Power and Control Autonomy for High-Speed Locomotion With an Insect-Scale Legged Robot. IEEE Rob. Autom. Lett..

[18] Nemitz M.P., Sayed M.E., Mamish J., Ferrer G., Teng L.J., McKenzie R.M., Hero A.O., Olson E., Stokes A.A. (2017). HoverBots: Precise Locomotion Using Robots That Are Designed for Manufacturability. Front. Robot. AI.

[19] Birkmeyer P., Peterson K., Fearing R.S. DASH: A dynamic 16g hexapedal robot. Proceedings of the IEEE/RSJ International Conference on Intelligent Robots and Systems (IROS).

[20] Hoover A.M., Steltz E., Fearing R.S. RoACH: An autonomous 2. 4 g crawling hexapod robot. In Proceedings of the IEEE/RSJ International Conference on Intelligent Robots and Systems (IROS).

[21] Zhu P., Peng H., Lu X., Guo M., Zhao G., Liu W. (2020). A steerable miniature legged robot based on piezoelectric bending actuators. Smart Mater. Struct..

[22] Ng C.S.X., Tan M.W.M., Xu C., Yang Z., Lee P.S., Lum G.Z. (2021). Locomotion of Miniature Soft Robots. Adv. Mater..

[23] Fan X., Dong X., Karacakol A.C., Xie H., Sitti M. (2020). Reconfigurable multifunctional ferrofluid droplet robots. Proc. Natl. Acad. Sci. USA.

[24] Xiao J., Xiao J., Xi N. Minimal power control of a miniature climbing robot. Proceedings of the IEEE/ASME International Conference on Advanced Intelligent Mechatronics (AIM).

[25] Kossett A., D’Sa R., Purvey J., Papanikolopoulos N. Design of an improved land/air miniature robot. Proceedings of the IEEE International Conference on Robotics and Automation (ICRA).

[26] Seok S., Onal C.D., Cho K.-J., Wood R.J., Rus D., Kim S. (2012). Meshworm: A peristaltic soft robot with antagonistic nickel titanium coil actuators. IEEE/ASME Trans. Mechatron..

[27] Kohut N.J., Hoover A.M., Ma K.Y., Baek S.S., Fearing R.S. MEDIC: A legged millirobot utilizing novel obstacle traversal. Proceedings of the IEEE International Conference on Robotics and Automation (ICRA).

[28] Pickem D., Lee M., Egerstedt M. The GRITSBot in its natural habitat-a multi-robot testbed. Proceedings of the IEEE International Conference on Robotics and Automation (ICRA).

[29] Pullin A.O., Kohut N.J., Zarrouk D., Fearing R.S. Dynamic turning of 13 cm robot comparing tail and differential drive. Proceedings of the IEEE International Conference on Robotics and Automation (ICRA).

[30] Rios S.A., Fleming A.J., Yong Y.K. (2016). Miniature resonant ambulatory robot. IEEE Rob. Autom. Lett..

[31] Rubenstein M., Ahler C., Nagpal R. Kilobot: A Low Cost Scalable Robot System for Collective Behaviors. Proceedings of the IEEE International Conference on Robotics and Automation (ICRA).

[32] Klingner J., Kanakia A., Farrow N., Reishus D., Correll N. A stick-slip omnidirectional powertrain for low-cost swarm robotics: Mechanism, calibration, and control. Proceedings of the IEEE/RSJ International Conference on Intelligent Robots and Systems (IROS).

[33] Zarrouk D., Fearing R.S. (2015). Controlled In-Plane Locomotion of a Hexapod Using a Single Actuator. IEEE Trans. Rob..

[34] Zarrouk D., Fearing R.S. Compliance-based dynamic steering for hexapods. Proceedings of the IEEE/RSJ International Conference on Intelligent Robots and Systems (IROS).

[35] Dharmawan A.G., Hariri H.H., Foong S., Soh G.S., Wood K.L. Steerable miniature legged robot driven by a single piezoelectric bending unimorph actuator. Proceedings of the IEEE International Conference on Robotics and Automation (ICRA).

[36] Wang G., Li C., Yuan T. (2017). Design and experiment of a small-scale walking robot employing stick-slip motion principle. Rev. Sci. Instrum..

[37] Zhang Y., Zhu R., Wu J., Wang H. (2022). SimoBot: An Underactuated Miniature Robot Driven by a Single Motor. IEEE/ASME Trans. Mechatron..

